# Eating and Control Styles Axis in Mentalisation-Based Psychotherapy in Eating Disorders: A Randomised Clinical Trial

**DOI:** 10.3389/fpsyt.2022.774382

**Published:** 2022-05-13

**Authors:** Moria Golan

**Affiliations:** Department of Nutrition, Tel-Hai Academic College and Private Eating Disorder Centre, Upper Galilee, Israel

**Keywords:** eating disorders, mentalisation, self-regulation, self-control, self-agency, eating style, tool

## Abstract

**Background:**

Clinicians need an instrument that helps their patients with eating disorders (ED) to explore their agent’s inner intentions and confront negative behaviour and control styles.

**Objectives:**

To assess the feasibility and impact of an eating and control styles axis (ECOSA) during the first 8 months of mentalisation-based psychotherapy with a community-based sample of ED patients.

**Methods:**

Six experienced therapists and their consecutively admitted patients were randomly allocated to the intervention and control groups. A total of 94 women, *M*_age_ = 24 were recruited between June 2020 and October 2021. Ninety completed it. Both groups received mentalisation-based psychotherapy, but only the intervention group used the ECOSA repeatedly. Therapists and participants were blinded to the study aims and hypothesis. Fidelity assessment was applied to ensure that the two groups differed mainly in terms of ECOSA usage.

**Results:**

The use of ECOSA, although less than advised, was reported as feasible. The effect size of the improvement in reflective functioning was larger than that of the control group and correlated significantly only in the intervention group with EDE-Q score (*r* = 0.46; *p* = 0.001).

**Conclusion:**

Although the study limitations: selective population, relatively small sample size and the lack of controlled confounder, the combined quantitative and qualitative results lend preliminary evidence for the validity and contribution of ECOSA as a possible instrument that may upgrade the clinician’s toolbox in the treatment of ED. A more rigorous study design is needed to explore the potential usage of ECOSA as a clinical tool to enhance mentalisation among people with ED.

## Introduction

Eating and control styles have a bidirectional relationship involving various areas associated with affective processing.

When people actively regulate (suppress) their emotions and affective responses, their amygdala shows reduced activity whereby regions of the prefrontal cortex (PFC) show increased activity (1). It works like a muscle. It must be built up over time and can become fatigued if overexerted. Decreased PFC activity causes a decrease in goal-directed decision-making and disrupts the dopamine reward circuit, both considered contributors to dysregulated eating behaviours (2).

Dietary restraint, fasting, food preoccupation, binging, and purging are disordered eating behaviours perceived as maladaptive avoidance-based coping strategies, providing distress escape methods e.g., avoidance, numbing and withdrawing, and harmful releases (3, 4). The distress escape methods are mediated *via* the behavioural activation system (BAS), the behavioural inhibition anxiety (BIA), and the fight-flight-freeze system (FFFS) which are parts of the emotional systems (5). The proportional activity of these sites has significant implications for several approach/avoidance patterns. Specifically, elevated behavioural inhibition and FFFS activity broadly increase vulnerability to anxiety and internalising disorders and facilitate avoidance of aversive and distressing stimuli, while the BAS elicits approach behaviours toward pleasant stimuli, and thus, is more associated with impulsivity and externalising disorders (6). Recent findings suggest a significant direct effect of affective lability and significant indirect effects of emotion regulation (ER) difficulties and distress intolerance on impaired ability to self-control and cease eating (7). When eating disorders (ED) serve as ER strategies, they are perceived as useful in the first instance, generating an initial reduction in emotion. However, they are maladaptive methods that trigger further negative emotional experience and reinforce negative beliefs and schemata; thus, a vicious cycle develops (8).

People with ED struggle with both controlling and out-of-control symptoms, which are sometimes profoundly confusing for the person. High self-control or overcontrolling have been related to anorexia nervosa (AN) and restricted eating in ED subtypes, often characterised with higher dopaminergic activity which strengthen the ventral striatal hypothalamic food-control circuity, enabling individuals to override normal hunger cues (9, 10). The out-of-control eating related to all ED subtypes with binge eating due to emotion-induced impulsivity (11) or self-regulation failures under stress are often reported by restrained eaters with binge eating disorder and bulimia nervosa (BN) (12). Among the latter, lower dopaminergic activity, which is associated with hedonic eating, based on pleasure rather than energy needs, facilitates intermittent binge eating episodes (13).

Transition between the various ED syndromes and behaviours associated with over or under control were suggested to be the result of the change in brain reward processing, which may alter food intake control circuitry and reinforce the individual’s ED behaviour (14, 15).

People with ED frequently fail to identify food restriction as overcontrolling behaviour and rationalise eating less and undernourishment as their healthy choice to exercise control (16). Similarly, overeating is frequently perceived as addressing individual’s desires rather than impulsivity. Moreover, the perception of recovery among sufferers of ED predominantly involves overcontrolling their bodies and eating, rather than reaching moderated eating and control styles. They are rarely aware of their impaired emotional self-regulation that underlies their symptoms.

Impaired ER, self-control, mentalisation, and self-agency, are reported as transdiagnostic dimensions in ED (17). Therapeutic strategies aim to target emotional processing difficulties by addressing poor understanding and perceived values in the individual’s needs and emotions to enhance individual self-agency (18). The associations among the dysfunctions in neural systems, deficits in neurocognition, maladaptive behaviours, and cognitions have prompted various interventions which focus on changes in cognitive and behavioural habits, enhancing cognitive flexibility, decision-making, inhibitory control (e.g., CBT), and improving self-regulation (e.g., DBT) to improve psychiatric symptoms (17–19). Psychoeducation and mentalisation-based psychotherapy were suggested as core elements in treating ED to enhance self-regulation and motivation for adequate self-care (20).

The capacity for mentalisation is thought to be a prerequisite for a good sense of agency about emotions and behaviour (21). The provision of mentalisation-based psychotherapy to patients with ED and expanding their understanding of the association between self-regulation, emotional-urgency, impulsivity, and self-control and the symptoms of ED were reported to be beneficial by these patients (22).

The importance of clarifying terminology in psychological science has been increasingly emphasised (23). Knowledge of how therapy and personal interaction change and shape the brain is vital in understanding how to best provide recovery to those who suffer from an eating-related pathology. Psychotherapy is still the most efficacious treatment for increasing the rational behavioural choice and reducing impulsivity associated with distress intolerance, in people with ED. Understanding the continuous axis presenting the control and eating styles may reduce blame and focus on obtaining self-agency in employing adaptive behaviours as a way to shape the dynamic brain again.

Various psychoeducational visual tools and strategies are suggested for people with ED to strengthen their key role in understanding and adopting a positive moderated coping style. Some of them focus on the emotional foundation of the disease beyond the symptoms (24, 25). However, the disorder has poor rates of remission and high levels of relapse. Current psychological interventions facilitate small change, with better interventions needed (26).

This study aims to address the gap in psychoeducational visual tools for the treatment of ED and translate brain research into practice as well as enhance the effectiveness of ED therapy. To the best of the author’s knowledge, there are no tools that integrate the eating and control styles to improve a person’s mentalisation, and hence, improve self-agency and step toward better choices and recovery from ED.

Eating and control styles axis (ECOSA) is a tool developed to improve awareness, acceptance, and evaluation of one’s own adaptive interoceptive emotional experience over exteroceptive feedback to achieve emotional validation, emotional self-efficacy, and self-agency. The focus is on clarifying the association between an individual’s over and/or under control coping styles in choice to gain control over highly upsetting, uncomfortable, and intolerable feelings and how they can influence the decision taken during the behavioural ambivalence in the various categories of ED (27). Its feasibility and impact were assessed in a pilot randomised controlled clinical trial.

**FIGURE 1:**
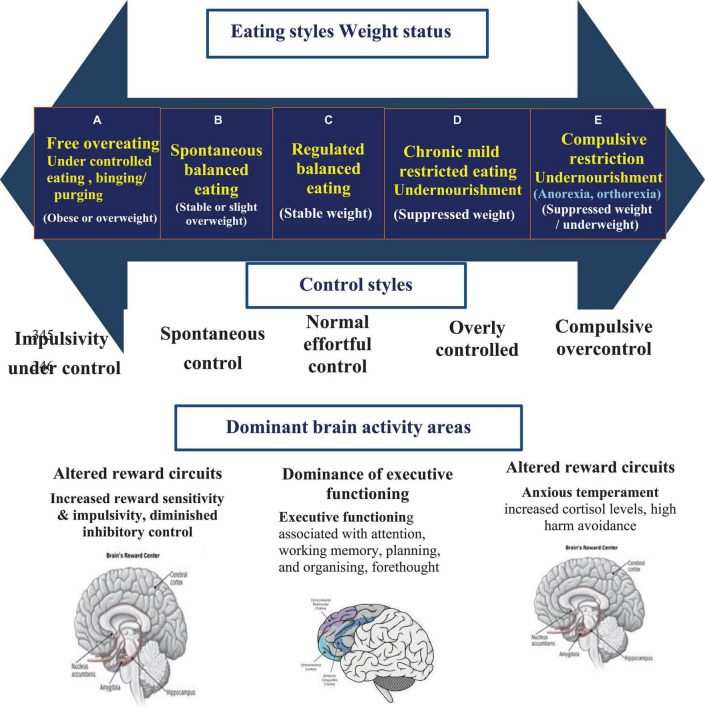
Eating and control styles axis (ECOSA) and the dominant brain activity.

The top part of the axis presents the eating styles and resulting weight statuses, the control styles are given below it, and the bottom part presents the type of decision taken – rational vs. emotional (Figure 1). On the left end of the continuous axis is the under-controlled eating group (A) which is often seen among people with BED and BN. On the right end of the continuous axis is the overcontrolling group, with rigid and restrictive behaviours (E) often seen among AN, orthorexia, and non-specific ED. More details were provided by Golan and Mozeikov (27).

As a feasibility study, our primary aim is to examine the possibility of incorporating the ECOSA with mentalisation-based eating disorders’ community-based treatment and assess the acceptability of the tool among therapists and their patients. The secondary aim is to assess the tool’s impact on treatment outcomes after 8 months compared to patients who received the same treatment without using this tool. Our first hypothesis is that most therapists will feel they benefited from the usage of ECOSA. The second hypothesis suggests that incorporating ECOSA into mentalisation based treatment will yield better outcomes regarding reflective functioning and ED’s symptoms.

## Methods and Measures

### Study Design and Ethics

The feasibility and effectiveness of ECOSA were assessed in a pilot randomised clinical trial. The preregistered NIH identifier is NCT04433663 (June 2020). Ethical approval for this study was granted by the Ethics Committee of Tel-Hai College. Patients and therapists received information about the study, and each provided informed consent. The study met the intent and requirements of the Helsinki declaration and Consort 2010 report checklist.

### Participants and Procedures

The participants of this study were recruited within a prospective longitudinal investigation. The clinical sample comprised of outpatients with ED and their therapists. Six experienced clinical psychologists (more than ten years in the field of ED treatment) in one community-based ED centre were randomly allocated to the intervention and control groups, using the Microsoft Excel (2010) randomisation function.

**FIGURE 2:**
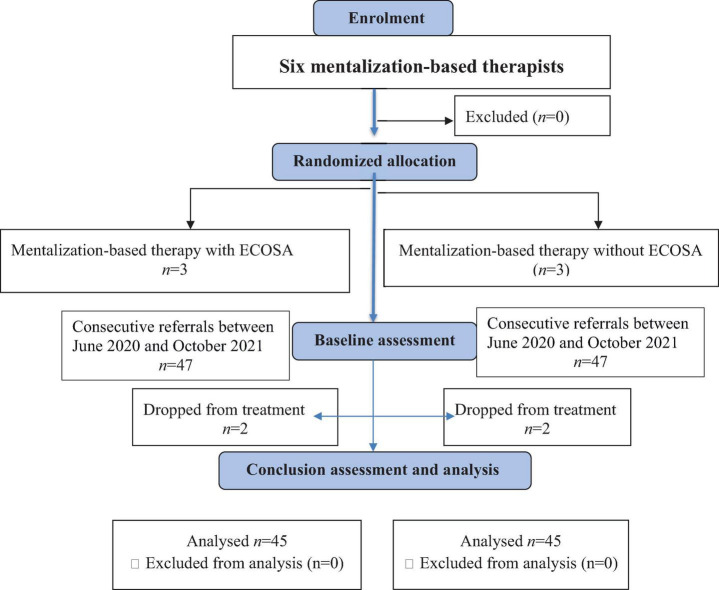
Study flow diagram.

All participants in the clinical sample were drawn consecutively from the new referrals to these therapists between June 2020 and October 2021. Patients were excluded from the study if outpatient treatment was deemed unsafe or if they had comorbidity of drug abuse. The sample (total of 94 patients) included 47 participants in each group, all women (Figure 2). The mean age of patients was 24.32 (SD = 3.2, range 17–29). All were previously treated in other ED units with a mean length of 3 months (range of 1–6 months). About 41% of participants in each group were diagnosed with BN, 11% with BED, 37% with AN, and less than 10% with Avoidant/Restrictive Food Intake Disorder (ARFID). Percentages of participants with major depression (75%) and/or borderline personality disorder (BPD) (30%) on the SCID-II (28) was similar in both groups. The overall sample had predominantly high socioeconomic status measured by the number of cars and rooms per person in the family.

Each therapist received a written consent from their agreeing patients to participate in the study and to record all sessions. Therapists and participants were blinded to the research arms and study aims. Patients in both groups received an individual mentalisation-based psychotherapy and assumed that the study is about mentalisation-based therapy outcomes (20). Only therapists randomly allocated to the intervention group received preliminary instructions on the ECOSA and its usage. Weekly mentalisation based supervision was provided to all therapist in the centre, with no reference to ECOSA. Therapists allocated to the intervention group signed on a confidentiality form about the usage of ECOSA to avoid contamination of the conditions, still they were blind to the study aims and arm allocation. Therapists and patients were not aware to the fact that some used the ECOSA, and some did not. Tool administration included explanation of the axis, identification of the client’s location on it and their underlining needs and perceptions and monitoring an example of change on the axis of one patient. Therapists were advised to expose participants to the tool at least twice a month in the first 3 months and subsequently at least once a month (about 11 times along the first 8 months of the treatment). Each therapist in the intervention group received a memo on their mobile phones once in 2 weeks with motivational enhancement sentences that reminded them of the advantages of ECOSA. For example: “With ECOSA, patient resistance to take responsibility, maybe an opportunity rather than a problem” or “ECOSA is one of your vehicles to deepen your patients” “self-agency and growth” or “You can demonstrate your patients’ journey toward moderating control using ECOSA.”

At the end of the study (after 8 months), each therapist allocated to the intervention group was interviewed by a research student (Appendix 1). All interviews were recorded and transcribed for qualitative analysis. Participants’ sessions were also recorded over the 8 months of treatment. Four randomised samples for each participant were checked for therapy-focused fidelity by two different psychotherapists.

### Measures

All participants underwent a standard assessment of ED on admission according to the DSM-5 criteria (29), including a semi-structured interview to measure comorbid disorders by an experienced psychologist (28). Participants in both groups completed the Hebrew validated Eating Disorder Examination Questionnaire (EDE-Q) (30) and the Reflective Functioning Questionnaire (RFQ((21) as part of a larger assessment battery. The EDE-Q is a 28-items self-report questionnaire assessing the core symptoms of ED and a wide range of associated pathology. The EDE-Q has four subscales and a global score: dietary restraint, eating concern, weight concern, and shape concern, each containing five to eight items. The responses to 22 items are rated using a 7-point forced-choice format (0–6), with higher scores reflecting greater severity. The remaining six items about the frequency of weight, shape, and purging techniques during the past 28 days require open, numerical responses. A cut-off of 4 on the global score is generally used as clinically significant (31). The global score is the average of the four sub-scales for men and women (32).

The Cronbach’s alpha in this study ranged between 0.82–0.90. Beyond the EDE-Q questionnaire at admission and follow-up, weight, binging, purging, and preoccupied thinking were monitored once a week.

The RFQ was developed as a brief, easy-to-administer screening measure to assess severe impairments or imbalances in mentalisation capacities. It includes eight items, scaled on a 7-point Likert-type scale. High values on this scale indicate high uncertainty regarding mental states; hence, difficulties with mentalising (33). The Cronbach’s alpha in this study ranged between 0.8–0.94.

To monitor patients’ exposure to the tool, each therapist received a monitoring table where they had to write their clients’ names, dates, and number of exposures of each researched patient to ECOSA. Feasibility was assessed by numbers of ECOSA exposures to each patient using a table that was provided to all intervention group’s therapist (Appendix 3), and by the qualitative final interviews with therapists. Data from these tables were collected every 2 weeks by the organization secretary.

### Data Analysis

The research student who collected the computerized data and the statistician were blinded to which group the information belong by naming the groups mentalisation 1 and 2. All analyses were conducted using SAS 9.4. The sample size was calculated prior to the study, using the mean and SD of the primary outcome score in the reflective functioning questionnaire (Appendix 2), it indicated the need for 26 participants in each group for 80% statistical power. Intention to treat analysis was performed, with missing values replaced with baseline values. Normality distributions and outliers for each outcome variable were examined before commencing analysis using the Shapiro–Wilks test. Summary statistics are reported as mean and standard deviation (SD) and in Appendix 4.

Baseline groups’ characteristics were compared using *T*-test for independent samples. Repeated measures two-way ANOVA analysis was used to assess the main effect of implementation status and the interaction between Group × Time. Pearson’s correlations were computed to assess the relationship between changes in ED symptoms and mentalising capacities. A *p*-value < 0.05 was used as the threshold for statistical significance in the testing of our hypotheses. To facilitate the interpretation of comparisons, omega squared effect sizes for ANOVA analysis and Hedges’ *g* for pairwise analysis were calculated for each significant result (34).

## Results

Data for 90 patients, 45 for each group of mentalisation-based therapists were analysed in the quantitative part, and 3 therapists that were randomly allocated to the usage of ECOSA (intervention group) were interviewed for the qualitative part.

### Feasibility

The data revealed that most therapists used ECOSA less than advised (about 11 times during the 8 months). The range of reported usage was 2–14 times per patient, with a mean of 7.1 ± 2.7. All three therapists allocated to the intervention group reported that they intend to use ECOSA in the future. The primary reason is that using this tool made it easier for people with ED to deeply relate with their control conflict. Therapists reported that “patients felt that they really understand and remember the control axis and related eating behaviours ‘and’ also found it effective in allocating their move on the axis along with the treatment.” “It served as a ‘progressing marker.”’ One therapist found it very useful when used alternately about 3–4 times during the first 3 months of the therapy. The other two therapists who used it less frequently said, “they did not want to be perceived as a grinder.” The barriers to intensive usage were: “it was hard to direct the conversation to the usage of the tool and thinking about it impaired the spontaneity” and “after using it 4 times the patient seems to internalise the concepts so why repeating myself?”

### Outcomes

At the baseline, no significant differences were found regarding mean age, ED behaviours, and perceptions measured by the global score of EDE-Q and mean RFQ scores (Table 1). During the first three to 5 months of treatment, two participants (∼5%) dropped out in each group (Figure 2).

**TABLE 1:** Baseline values of age, FRQ and EDEQ in both groups (means, SD, *p*, effect size, and 95% CI).

Baseline	Intervention *n* = 47		Control *n* = 47		*p-*Value^1^	Hedges’ *g* effect size (95% CI)
	Mean	SD	Mean	SD		
Age	24.4	0.86	3.1	24.2	2.9	0.07 (−0.35 to 0.48)
FRQ	2.2	0.83	0.6	2.3	0.6	0.16 (−0.25 to 0.58)
EDEQ	4.6	0.65	1.1	4.5	1.1	0.09 (−0.32 to 0.50)

Repeated measure ANOVA demonstrated statistically significant improvement with a large effect size in ED symptoms (EDE-Q) among both groups (large effect size) with no Group × Time interaction (Table 2). At 8 months from baseline, mean participants’ BMI was above 20 (within the normal range) and participants reported no vomiting and less than once a week of overeating in both groups.

**TABLE 2:** Change in FRQ and EDEQ scores in both groups (means, SD, *p*, effect size, and 95% CI).

Index	Time	Group 1 *N* = 45		Group 2 *N* = 45		Effect	*F* (df)^1^	Effect size^2^ (95% CI)
		Mean	SD	Mean	SD			
FRQ	T1	2.2	0.6	2.3	0.6	Group	30.28 (1,88)***	0.25 (0.11–0.39)
	T2	0.2	0.2	1.2	0.6	Time	671.39 (1,88)***	2.28 (1.90–2.65)
						Group × Time	74.48 (1,88)***	1.95 (1.45–2.45)
EDEQ	T1	4.6	1.1	4.5	1.1	Group	1.22 (1,88)	0.002 (0.00–0.09)
	T2	1.3	0.5	1.2	0.4	Time	675.65 (1,88)***	3.82 (3.33–4.32)
						Group × Time	0.09 (1,88)	0.08 (−0.33 to 0.50)

*^1^Repeated measures ANOVA.*

^2^Omega squared for ANOVA and Hedges’ g for pairwise. ***p < 0.001.

The reflective functioning (FRQ) analysis revealed a statistically significant superiority in the intervention group improvement with a large effect size of Group × Time interaction, although in both groups a statistically significant improvement with a large effect size was observed (Table 2).

**FIGURE 3:**
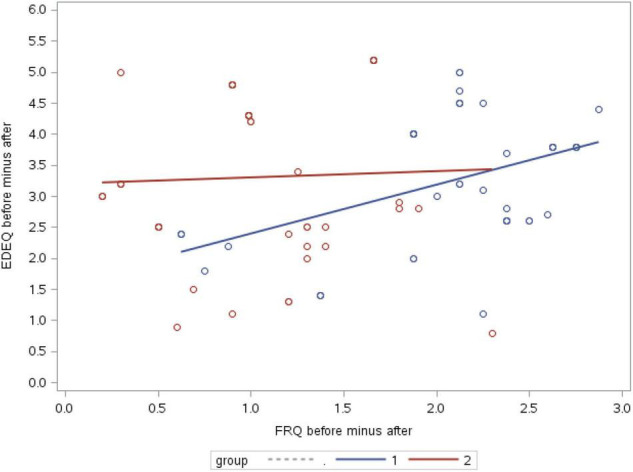
Correlations between changes in RFQ scores and changes in EDE-Q scores (1, intervention group; 2, control group).

Correlations between changes in RFQ scores and changes in EDE-Q scores (Table 3 and Figure 3) showed a statistically significant positive correlation only in the intervention group (*r* = 0.46; *p* = 0.001), conveying that ED symptoms improve with improvements in reflective functioning.

**TABLE 3:** Pearson correlation between improvement in EDEQ and improvement in FRQ scores, by group.

	*r*	*p*
Intervention group	**0.458**	**0.001**
Control group	0.039	0.79

*The significant result is bolded.*

## Discussion

Difficulties in managing negative emotions and urgency represent transdiagnostic risk factors in various psychological disorders, including ED (16, 35). Although the behavioural manifestations of these characteristics may oscillate, the core psychopathology is expressed in the relative balance of under-control and over-control and its effect on body weight and eating behaviour due to fear of losing self-control (10).

The current study assessed the feasibility and impact of using ECOSA (27), a continuous axis to clarify the different eating and control patterns (under or over control) and address impaired mentalisation and self-agency in respect to these patterns and ED symptoms in community-mentalisation based treatment for ED patients.

The qualitative assessment confirmed the feasibility of ECOSA. It was described by therapists as a convenient and feasible tool to clarify the association between people’s behaviour and control styles. Moreover, it was found to be an effective tool for assessing the progress in control and eating patterns. However, some may perceive the repeated usage as oppressive, mainly those that prefer unstructured and spontaneous journeys.

Eating and control styles axis’ impact on treatment outcomes was evaluated using a pilot, blinded, randomised, and controlled trial with 6 participating therapists and 94 patients. At baseline, the demographic and clinical characteristics as well as the values of the two primary outcomes of EDE-Q and RFQ were not statistically different between intervention and control groups, as opposed to previous warnings in the literature, indicating that baseline non-equivalence between intervention groups was a significant common bias in clinical trials (36). Moreover, the global EDE-Q and RFQ scores were comparable to those reported by others with a clinical population (31, 33).

Premature termination of ED treatment among outpatients is a significant problem in treating ED, particularly in adults (37). In our study, less than 5% of participants terminated the treatment prematurely. It is below the range of reported values in community-based clinical ED population studies (38, 39) and typical in the described clinic due to intensive reaching-out and motivational enhancement approach, emphasising the client’s autonomy, while reflecting on the prices of surrendering to the ED rules.

Mentalisation-based treatment with or without ECOSA produced statistically significant improvement in mentalisation with large effect size, in both groups, reaching a non-clinical population’s values (33).

Nevertheless, analysis revealed large effect size of the intervention group superiority expressed *via* the statistically significant interaction Group × Time.

After 8 months of treatment, a statistically significant improvement in ED symptoms with a large effect size was observed in both groups. The mean score of the global EDE-Q at the 8 months follow-up reached the normal range (30, 35), although the treatment journey continued, addressing the core emotional foundations under ED. Nevertheless, only in the intervention group it was statistically and significantly correlated with the improvement in the reflective functioning score, suggesting that the improved outcomes in ED symptoms were more related to the improved mentalisation when the ECOSA was used.

### Strength and Limitations

The routine clinical research setting performed habitually in our institute is one of this naturalistic study strength. The longitudinal data collection with standardised baseline admission process includes diagnostic as well as narrative interviews. These are followed by completion of assessment battery including various validated scales as well as routine clinical supervision and fidelity assessment process. A pilot randomised controlled clinical trial with a blinding condition, intention to treat analysis, and fidelity assessment were applied in this study, to ensure that the two groups differed mainly in the presence and absence of ECOSA usage. The effect sizes provide a valid indication of the strength of the relation beyond the issue of power and significance. The combination of quantitative and qualitative measures applied in this study provide a comprehensive understanding of the feasibility and impact of ECOSA usage and knowledge about some of the possible barriers.

The findings of this study need to be interpreted in the context of some key limitations. First, the patient sample was drawn from one community-clinic with high socio-economic status, thus, it is a selective sample, and the results may not be generalisable. Although no differences were found between groups in baseline participants’ characteristics, some confounders which were not measured might affect the results, for example, therapists’ and patients’ motivation, cognitive flexibility, and more. Due to relatively small sample size, regression analysis was not possible; thus, there is a risk of more significant false-positive effects sizes and producing the predicted contribution of ECOSA (40). The use of self-administered instruments, which are often biassed, is another limitation of this study. However, as they were used similarly in both groups, the computerised desirability bias is smaller and the impact on the comparison outcomes is reasonable.

Despite the study limitations it lends preliminary evidence for the validity and contribution of ECOSA as a possible instrument that may upgrade the clinician’s toolbox in the treatment of ED. This report addresses the gap between theory and practice in managing ED regarding the transdiagnostic symptoms – impaired ER, self-control, impaired mentalisation, and self-agency. Improved mentalising skills could lead to better treatment outcomes and lower relapse rates. However, additional studies would be needed to validate this tool further. Future study may benefit from a greater sample size and more rigorous statistical methods. The field will benefit from assessing mediating or confounding factors such as cognitive flexibility, for example, that might influence therapist and patients’ responsiveness as well as applicability of the tool. Moreover, understanding of the biological patterns underlining the various options on the axis may extend the integration between theory and practice in managing ED.

## Conclusion

Results of this pilot study lend preliminary evidence for the vital role of a mentalisation tool in ED treatment and warrant consideration of more rigorous study designs to explore the potential usage of clinical tools to enhance mentalisation among people with ED.

## Data Availability Statement

The raw data supporting the conclusions of this article will be made available by the authors, without undue reservation.

## Ethics Statement

The studies involving human participants were reviewed and approved by the Tel-Hai Academic College IRB. The patients/participants provided their written informed consent to participate in this study.

## Author Contributions

MG developed the ECOSA tool, designed the feasibility, wrote the article, and approved the submitted version.

## Conflict of Interest

The author declares that the research was conducted in the absence of any commercial or financial relationships that could be construed as a potential conflict of interest. The handling editor declared a shared affiliation, though no other collaboration with the author at the time of the review.

## Publisher’s Note

All claims expressed in this article are solely those of the authors and do not necessarily represent those of their affiliated organizations, or those of the publisher, the editors and the reviewers. Any product that may be evaluated in this article, or claim that may be made by its manufacturer, is not guaranteed or endorsed by the publisher.

## Figures and Tables

**Table:** 

	Intervention group								
Month patient	1	2	3	4	5	6	7	8	Total
1	2	1	0	1	0	1	1	0	**5**
2	1	0	0	0	1	0	1	0	**2**
3	1	2	1	1	1	1	1	1	**9**
4	1	2	0	1	1	0	1	1	**7**
5	3	1	1	1	1	1	1	1	**10**
6	1	0	1	0	0	1	0	0	**3**
7	1	1	2	1	0	1	1	0	**5**
8	3	2	2	2	1	1	1	2	**14**
9	3	1	1	1	2	1	0	0	**9**

	Mean 7.11 ± 3.7								

10	2	2	1	1	1	1	1	1	**10**
11	1	2	0	1	1	1	1	1	**9**
12	2	1	0	1	1	1	1	1	**8**
13	2	1	1	1	2	1	1	1	**10**
14	2	1	1	1	1	1	1	1	**9**
15	2	1	1	1	1	1	1	1	**9**
16	2	1	1	1	1	1	1	1	**9**
17	3	1	1	1	1	1	1	1	**10**

	Mean 9.25 ± 0.7								

18	1	1	0	1	0	1	0	0	**4**
19	3	0	1	0	1	1	0	0	**6**
20	0	1	2	0	1	0	0	0	**4**
21	0	1	1	0	1	1	0	0	**4**
22	2	2	1	0	1	0	0	0	**6**
23	2	0	1	0	1	0	1	0	**5**
24	3	1	2	1	0	0	0	0	**7**
25	2	1	1	0	1	0	0	0	5

	Mean 5.25 ± 1.0								
									

**Table:** 

Group	Variable	*N*	Mean	SD	Median	Minimum	Maximum
**Skewness**							
1	FRQ_BE	45	2.2	0.6	2.4	0.9	3.0
	FRQaft	45	0.2	0.2	0.1	0.0	0.6
2	FRQ_BE	45	2.3	0.6	2.4	0.7	3.1
	FRQaft	45	1.2	0.6	1.1	0.0	2.6
**Kurtosis**							
1	FRQ_BE	45	4.6	1.1	4.6	2.5	6.2
	FRQaft	45	1.3	0.5	1.2	0.4	2.5
2	FRQ_BE	45	4.5	1.1	4.2	2.4	6.0
	FRQaft	45	1.2	0.4	1.1	0.6	2.2

## Appendix

### Appendix 1: Interview Guide for Semi-Structured Interviews

1. How long have you been working as a psychotherapist in this service?
2. What is your professional background?
3. Overall, how did you like the ECOSA tool?
4. Do you intend to use it when the current project will stop?
5. What did you feel was working well in using it?
6. You used it with ____(how many clients?) How many of them you felt really benefited of it?
7. Could you please share with us your table and see how many times in average you used the tool with each of your client?
8. For what purpose you found the tool most beneficial, and would you please describe in which months of the treatment was it?
9. What do you feel you could be doing differently?
10. Where there any specific barrier or challenges you faced when using the ECOSA? Any issues that you came across?
11. Closing questions: Is there anything you would like to add?
12. Thank you for your time.
